# Learning non-adjacent rules and non-adjacent dependencies from human actions in 9-month-old infants

**DOI:** 10.1371/journal.pone.0252959

**Published:** 2021-06-09

**Authors:** Helen Shiyang Lu, Toben H. Mintz

**Affiliations:** 1 Department of Psychology, University of Southern California, Los Angeles, CA, United States of America; 2 Department of Linguistics, University of Southern California, Los Angeles, CA, United States of America; Max-Planck-Institut fur Kognitions- und Neurowissenschaften, GERMANY

## Abstract

Seven month old infants can learn simple repetition patterns, such as *we-fo-we*, and generalize the rules to sequences of new syllables, such as *ga-ti-ga*. However, repetition rule learning in visual sequences seems more challenging, leading some researchers to claim that this type of rule learning applies preferentially to communicative stimuli. Here we demonstrate that 9-month-old infants can learn repetition rules in sequences of non-communicative dynamic human actions. We also show that when primed with these non-adjacent repetition patterns, infants can learn non-adjacent dependencies that involve memorizing the dependencies between specific human actions—patterns that prior research has shown to be difficult for infants in the visual domain and in speech. We discuss several possible mechanisms that account for the apparent advantage stimuli involving human action sequences has over other kinds of stimuli in supporting non-adjacent dependency learning. We also discuss possible implications for theories of language acquisition.

## Introduction

Many events that humans and other organisms experience involve temporally ordered sequences. These include visual events, such as watching agents engaging in actions, and machines carrying out functions, as well as auditory events, such as hearing a sequence of words in a spoken sentence, or sounds within words, or even notes in a piece of music. In many cases, these events contain regularities in which certain elements within an event predict certain others. For example, in the action of hammering a nail, the agent first moves the hammer away from the nail, and then forcefully brings the hammer into contact with the nail. In the English present progressive, the copula, *is*, is followed by a verb with the inflection -*ing*, for example, …***is*** *bak*-***ing*** …. Through experience, individuals learn about aspects of these regularities, and, once noticed, can use them to generate new knowledge, either explicit, such as the understanding of an artifact’s function, or implicit, such as the knowledge of the grammatical rules of one’s native language(s). Substantial areas of cognitive development are devoted to understanding the processes by which experience leads to knowledge, and how these processes may be guided by more specialized or more general learning mechanisms. The study presented here is part of an endeavor to understand the very first steps of these processes. It address the questions: what kinds of regularities do infants detect when they experience temporally sequenced events? How do they generalize those regularities and use those generalizations to make predictions about other events? The answers to these questions are important for constraining theories of cognitive development, as they provide evidence about the kinds of representations infants have available as the input to further learning.

These questions have been widely investigated with respect to regularities involving co-occurring adjacent items. Numerous behavioral studies have shown that infants can track adjacent co-occurrence statistics in artificial and natural languages [1–4], as well as in musical tones [5]. Neurophysiological studies suggest that even neonates track adjacent co-occurrence statistics [6]. And other species, such as monkeys [7] and rats [8], have also been shown to be able to track adjacent co-occurrence statistics in human speech. Human neonates have also been shown to detect adjacent co-occurrence patterns in visual stimuli [9]. Extracting regularities involving adjacent items thus appears to be quite robust, and in some cases, not specific to humans.

However, less is known about infants’ ability to detect and learn from regularities involving non-adjacent items, yet these kinds of regularities are also ecologically important [10, 11]. For example, start and end states of a goal-directed action sequences may be related, even when different intermediate actions are implemented on the way from the beginning to the goal. In the linguistic example discussed earlier, the grammatical dependency between *is* and -*ing* is non-adjacent, and there can be considerable variability even in the number of intervening items (e.g., …***is*** *energetically bak*-***ing*** *bread* …). Understanding infants’ ability to detect and learn from regularities in non-adjacent elements is therefore critical to a comprehensive understanding of infants’ broader ability to learn from regularities in temporal sequences, across domains.

This paper focuses on infants’ processing of two different types of non-adjacent dependencies. The first type, which we call *ABA* dependencies, involves the repetition of an item across one intervening element. The critical pattern is non-adjacent repetition, where the non-adjacent items are identical. The second type we call item-specific dependencies (*a*X*b*), which involves a non-adjacent relationship between two specific items, *a* and *b*. We call these item-specific dependencies simply non-adjacent dependencies (NADs), as this is how the literature typically refers to them.

There are two distinct bodies of research that have started to map out the learning territory regarding these two types of non-adjacent regularities. As we overview in the next section, apparent differences have emerged from these studies with respect to the age at which infants detect the two types of non-adjacent dependencies, and the type of stimuli from which they can learn. Questions thus arise whether the same mechanisms are responsible for both types of non-adjacent dependency learning, or whether they are governed by different mechanisms with different developmental trajectories and operating principles. While we do not provide a definitive answer here, we believe this study contributes new insights into these questions, by bringing together these typically distinct lines of research in two behavioral experiments with infants. In the remainder of the introduction we provide a brief summary of the findings from the literature on *ABA* and NAD learning that motivate the current study.

### Learning *ABA* repetition rules

Seven-month-old infants have been shown to learn simple repetition patterns, such *ga-ti-ga*, or *ga-ti-ti*, and detect those patterns in a different set of syllables (e.g., *we-fo-we* or *wo-fe-fe*), indicating that they learned a generalization about the syllable repetition patterns—*ABA* or *ABB*, respectively [12].

To address the generality of this mechanism, infants’ ability to detect adjacent (*AAB* or *ABB*) and non-adjacent (*ABA*) repetition patterns has been explored in other domains, such as visual images and non-linguistic sounds. For example, Marcus, Fernandes, and Johnson [13] found no evidence that 7.5-month-olds could learn *ABA* or *ABB* dependencies in non-speech sounds (but see [14] for evidence of learning in 4-month-olds), but if they learn the rules in the speech domain, they can transfer the rules to non-speech sounds. In visual sequences of shapes, Johnson et al. [15] found no evidence that 8- or 11-month-olds could learn *ABA* rules, and only the older infants could learn *ABB* and *AAB* rules. The 8-month-olds showed an ability to detect adjacent repetitions in some cases, but they did not appear to have learned where within the sequence the repetition occurred. Thus, although infants can distinguish adjacent from non-adjacent repetition patterns in non-speech and non-auditory domains, the ability does not seem to be as robust as it is in speech. The particular situations that seem to be challenging in the visual domain involve *ABA* learning trials—that is, those that involve non-adjacent repetition patterns—suggesting that infants’ ability to detect these patterns in the visual domain is lacking in the age range tested.

However, other studies with 7-month-olds that used different methods have found evidence of repetition rule learning, including the learning of *ABA* patterns, with images of familiar objects: cats and dogs [16], and human faces [17]. The differences in results from these studies and those using shapes [15] could be the result of at least two important differences in the experimental designs. First, as just mentioned, in the experiments where infants learned *ABA* patterns, the stimuli were familiar categories. Second, the stimuli were presented such that infants could see the entire array of images concurrently. The visual stimuli appeared one at a time, from left to right, but then remained on the screen, with the entire set of three concurrently available for nearly one second. Thus, in contrast with Johnson et al. [15], the stimuli in these experiments were images of familiar categories that infants could see simultaneously. The familiarity of the categories could result in more reliable memory encoding and retrieval, thus facilitating learning. Moreover, in the concurrent presentation method, infants were able to visually inspect the entire sequence, which provided a greater opportunity for infants to notice the repeated items, while reducing memory and attentional load compared to sequential presentation. In other words, the learning problem was one of learning associations between entities in the spatial domain, rather than learning associations, via memory, in the temporal domain. The demands on memory and attentional resources are arguably much reduced in the former [18].

Taken together, the experiments just reviewed suggest that memory and attentional resources may be limiting factors for infants when learning *ABA* patterns (and NADs more generally). In addition, there appears to be an advantage for speech in *ABA* learning [12] over non-speech sounds [13] and visual stimuli [15], at least when the stimuli are temporally sequenced. Recently, some researchers have offered a broader explanation of the apparent advantage for speech, proposing that repetition rule learning—including learning the more challenging *ABA* patterns—is facilitated when the stimulus involves a communicative signal in general, of which speech is one [19]. In the visual domain, Rabagliati et al. [19] habituated 7-month-old infants to *ABA* and *ABB* rules involving handshapes from American Sign Language (ASL). Prior to habituation, one group of infants viewed a short video in which one actor used ASL gestures to communicate with another actor and the infant, and the other actor responded in speech. Two other groups saw either a short video in which two actors simultaneously produced the same gesture sequence but were not facing towards each other or the infant, or they saw no pre-habituation video at all. Infants in those two groups failed to learn the repetition rules from sequentially presented gestures during the subsequent habituation phase. Infants learned the rules only if they were first primed to interpret the gestures as communicative. The authors argued that the pattern of results supports the hypothesis that repetition rule learning is specialized for the domain of communicative signals. Given the results just reviewed [16, 17], such a specialization, if it exists, must apply only for temporally sequenced stimuli. However, in Experiment 1, we show that 9-month-old infants can learn non-adjacent repetition rules (*ABA* patterns) from non-communicative, temporally sequenced visual stimuli.

### Learning NADs

#### The auditory domain

Historically, research into item-specific NAD learning has been rooted in issues involving language acquisition. In their seminal study, Santelmann and Jusczyk [20] showed that 18-month-old English learners differentiated between ungrammatical sentences in which there was a violation between the auxiliary verb and main verb inflection (e.g. *the baker ***can*** *bak****ing*** *bread*), and their grammatical counterparts (e.g., *the baker* ***is*** *bak****ing*** *bread*). This demonstrated that young English learners represent some non-adjacent dependencies in their native language. Santelmann and Jusczyk [20] also found no evidence that 15-month-olds detected the same violations of non-adjacent dependencies. Gómez [21] trained 18-month-olds on an artificial language and showed that they differentiated grammatical strings from ungramamatical strings that violated the non-adjacent dependency patterns. For example, when familiarized to trigrams like *pel X jud*, *rud X jic* where the word in the *X* position varied across sentences, 18-month-olds listened longer to subsequent test sentences that violated the dependency (e.g., *pel X jic*) from those that did not, supporting Santelmann and Jusczyk’s findings [20]. Moreover, using the same artificial language procedure Gómez and Maye [22] demonstrated that 15-month-olds also detected violations of non-adjacent patterns, but found no evidence of this ability in 12-month-olds. In contrast, Marchetto and Bonatti [23] reported evidence of NAD learning in 12-month-olds. However, their findings are difficult to interpret since, in their experimental design, ungrammatical test strings also violated regularities in the items at the edges of the trigrams. Specifically, an edge position in the trigram had an item in an unattested position—an item that occurred only in middle positions during familiarization occurred in an edge position in testing—thus breaking the positional coherence. This created a confound with the NAD violation. Thus, in our view, clear behavioral evidence of NAD learning is absent in infants before 15 months. Moreover, in artificial language studies, NAD learning was shown to be sensitive to certain distributional properties of the intervening item: Without the high variability of the *X* words across trigrams, Gómez and colleagues failed to find evidence of NAD learning [21, 22]. Thus, in contrast to learning adjacent dependency patterns [1–9], NAD learning appears to be much less robust, across domains and species [24].

It is not surprising that pattern detection and learning is different for adjacent and non-adjacent patterns. Linking adjacent items requires minimal memory resources (although resources are required to store the link), and the relationship itself is quite restricted, pertaining only to the next (or previous) item. In contrast, detecting non-adjacent relationships requires holding one item in working memory as more items are processed, then linking that item to a subsequent (non-adjacent) item. Beyond this increase in resource demands, the computational problem increases because there are multiple non-adjacent relationships that the learner could consider: While adjacency is limited to one position, non-adjacency is bounded only by the length of the sequence. Moreover, there is evidence that learners compute adjacent patterns even as they are learning non-adjacent ones [25, 26], further increasing resource demands.

While behavioral evidence of NAD learning in younger infants is lacking, it is important to note that researchers using an artificial language like that in [21, 22] found neurophysiological evidence of NAD learning in 3-month-old infants [27]. The discrepancy between the equivocal behavioral evidence of NAD learning in 12-month-olds and the neurophysiological evidence in 3-month-olds could be the result of developmental changes in capacity [14], perhaps indicative of a U-shaped developmental process. Or, it could be that the mechanisms involved in NAD learning are in place from at least three months, but the representations involved are not sufficient to drive overt behavior [18]. Taken together, the infant literature suggests that detecting non-adjacent dependencies is more difficult and perhaps more fragile compared to processing adjacent dependencies. This is confirmed by many artificial language experiments with adults. For example, as with infants, Gómez [21] found that adults required high variability in the middle position of a trigram in order to learn the dependency between the first and last words (see also [25]). An additional property of many successful demonstrations of NAD learning in adults, such as [21, 25], is that the trigrams with the NADs were presented as discrete, pre-segmented sequences, with 750ms of silence between each NAD trigram. When word sequences with the same statistical properties as in [21] were presented in a continuous sequences, adults did not learn the NADs [26]; similar learning failures in continuous sequences were found at the syllable level [28]. Some researchers even theorize that humans are constrained to learn NADs only when the non-adjacent items are at the edges of sequences, as defined by brief silences [29]. Importantly though, other types of edge or boundary cues appear to facilitate NAD learning, such as top-down structural cues [30] and rhythmic cues [31]. Indeed, in those studies [30, 31], adults learned NADs without silences at the edges of the NADs, and with minimal variability between only three items in the middle position. Furthermore, Newport and Aslin [32] showed that when the non-adjacent elements are perceptually similar, and contrast with the intervening item—for example, when the non-adjacent segments are both consonants with a vowel intervening, or vice-versa—then adults can learn those dependencies from a continuous speech stream, but not when they do not share those similarities. Similar results were found with adults for musical pitches [33] as well as non-musical, computer alert sounds [34]. Taken together, for infants and adults, NAD learning from speech and other auditory stimuli appears to be much less stable compared to learning adjacent co-occurrence patterns, and much more dependent on properties of the stimulus that are unrelated formally to the dependencies.

#### The domain of visual human action

Other studies have examined NAD learning in domains that are perceptually much more distinct than speech and music, in particular the domain of visually presented human action. Many human actions are parsed as temporally ordered sequences of smaller actions or movements [35, 36], that are hierarchically structured, and where relationships between non-adjacent elements may be important (e.g., start states and goals/end states; [37]). Prior research has shown that adults [38] and infants [39] can segment continuous streams of dynamic human motions into units based on adjacent statistical dependencies. In studies that motivate the methodology for the experiments proposed here, Endress and Wood [40] exposed adults to videos of animated human avatars carrying out various actions (e.g., raising a knee, twisting the torso, bowing). In one experiment, adult participants saw a continuous sequence of action-triplets—sub-sequences of three actions—where the first and final action of each triplet implemented an NAD, and where the middle action alternated between three different actions. Endress and Wood [40] showed that adults acquired the action NADs, and this was replicated and extended by Li and Mintz [41] and Lu and Mintz [42]. Thus, learning NADs in visual sequences of human action seems to be more robust, at least for adults, compared to auditory sequences [33, 34], including speech [32]. Specifically, learning succeeds even when dependent non-adjacent elements are no more similar to each other than they are to the intervening element, and it succeeds with minimal item variability in the intervening position. Based on the strength of visual human action sequence in supporting NAD learning in adults, in Experiment 2 we tested 9-month-old infants’ ability to learn NADs from temporally sequenced human actions.

We are aware of one other study that tested NAD learning in visual sequences in infants. A recent study by Bettoni, Hermann, Brady, and Johnson [43] tested NAD learning in sequences of geometric shapes and arrays of dots. They found evidence of NAD learning in 13- to 15-month-olds, but not 9- to 12-month-olds. Interestingly, the elements that were part of the NADs were perceptually similar—simple shapes—and they contrasted perceptually with the intervening middle items—arrays of dots—much like in the non-speech auditory studies where NAD learning was successful [32–34]. This property might have supported NAD learning in the older infants, as it did in the auditory domain with adults, but it apparently was not sufficient to support learning in the younger infants.

### Research questions and approach

To recap, studies of *ABA* repetition rules in temporal sequences have suggested that infants’ repetition rule learning is specialized for communicative domains in vision and in speech, and is not otherwise engaged in processing auditory or visual stimuli. When the sequences are communicative, infants show sensitivity to *ABA* patterns as young as 7-months, but otherwise have not been shown to detect them even at 11 months [15, 19]. Infants’ NAD learning in speech appears to be even more limited. The earliest age at which learning has been reported in a behavioral study is 12 months [23], but, as noted earlier, with potentially problematic stimuli. Evidence from 15-month-olds’ successful NAD learning indicates that non-adjacent dependencies are not detected unless there is a high degree of variability in the middle position [22]. Even for adults, properties of the stimulus greatly influence NAD learning in speech. However, sequences of visual human actions appear to support both *ABA* and NAD learning in adults [40–42], and to support learning adjacent dependencies in infants [39]. It seemed plausible that visual human actions could support learning non-adjacent dependencies in infants. To our knowledge, there has been no prior research on non-adjacent dependency learning of visual action sequences in infants.

With these facts in mind, we set out to test whether we could find evidence of visual *ABA* learning and NAD learning in infants using visual sequences of human actions. To test this we carried out two behavioral habituation experiments with 9-month-old infants. In Experiment 1, we tested whether 9-month-olds can learn *ABA* rules, and in Experiment 2, we asked whether infants can learn NADs. In each case, the motivation for the experiments was our speculation that the apparent limitations and constraints on infants’ ability to compute the relevant structures in past experiments was due, in part, to infants’ ability (or lack thereof) to adequately encode the stimuli, rather than a limitation on computation. Some stimuli might provide richer representations than others, resulting in a better encoding and retrieval process for sequential pattern learning. Human actions are dynamic, and involve familiar and ecologically important entities. These factors could increase infants’ attention to the stimuli, and could cause them to be encoded more deeply. In addition, there is evidence that action observation results in activation of infants’ motor areas, as well as other areas specialized for processing biological motion [44–46], which could also result in richer encoding, and as a consequence a greater chance of retrieval compared to other types of visual stimuli. Moreover, some theories posit that the activation of the motor system in perception plays a role in perceptual prediction [47], which could further enhance processing of sequential information.

## Experiment 1

Infants under a year of age appear, in general, to be challenged when it comes to learning visual *ABA* rules in temporal sequences [15, 19], except when given special preparatory priming [19]. We speculated that stimuli that yielded richer representations would facilitate learning by enhancing infants’ ability to encode and process the sequential input. Given the relative robustness of dynamic human actions in facilitating NAD learning in adults [40, 41], we decided to test this hypothesis by testing whether infants can learn *ABA* rules in this stimulus domain. We examined 9-month-old infants’ sequential rule learning (e.g., *ABA* and *ABB*) with visual human actions—a domain combining both human forms and movements. If infants are able to learn sequential rules from visual human actions, then it suggests that infant rule learning mechanisms are influenced by factors other than the communicative function of the stimuli.

### Method

#### Participants

Infants were recruited from the Greater Los Angeles Area by emails and phone calls. The contact information were generated from a database of parents who had expressed interests in having their children participate in research after seeing our advertisements on Facebook. Parents gave written informed consent before infants started the experiment. At the end of the experiment, we gave the child a t-shirt or a small toy as a token of appreciation. We tested 18 full-term infants between 8.5 and 9.5 months of age (*M* = 9.0 months). Six additional infants were tested but not included in the analysis due to fussiness (*n* = 5), and premature birth (*n* = 1). The sample size was determined based on prior research investigating infants’ rule learning with visual stimuli using a similar method [15, 16]. The protocols for all of the experiments reported in this paper were approved by the Institutional Review Board at the University of Southern California.

#### Apparatus

Infants sat on their parents’ laps in a dimly lit room, with a 50-inch screen in front of them. Parents wore view-obstructing glasses and were instructed to not interact with their infants during the experiment. The experimental materials were presented using the software Habit 2.2.4 [48] installed on an HP EliteOne 800 computer running Windows 7. The stimuli were presented on the screen in front of the infant. An experimenter, blind to the stimuli that infants were viewing, observed the infant via a video feed in a separate control room and live-coded when infants looked at and looked away from the stimulus display screen.

#### Stimuli

**Fig 1:**
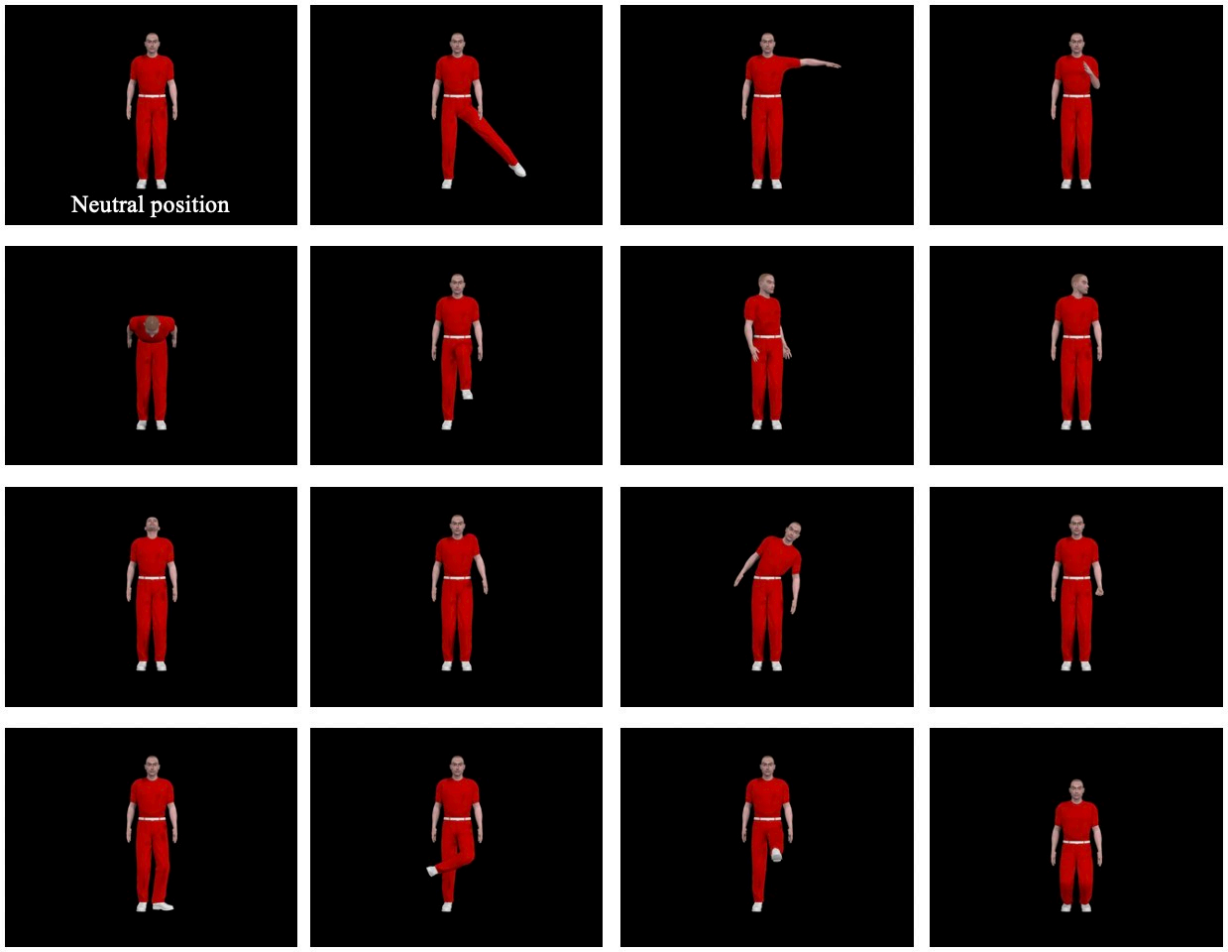
Human action clips used in Experiment 1 and 2. All clips were used in Experiment 2, whereas only 12 of them were used in Experiment 1. Each image is the midpoint of the action clip that depicts the largest movement within the entire clip, which starts and ends with the human avatar in a neutral, upright position with arms at the sides and head facing forward (labeled *Neutral* in the figure). Infants saw only one action clip presented on the screen at a time, and action clips within a triplet played sequentially with no pause in between.

Habituation and test materials were human action triplets similar to those used in [40] and [41]. Each action triplet was composed of a sequence of three action clips. Each clip within a triplet lasted 0.6 second, and started and ended with the animated human avatar in a neutral, upright position with arms at the sides and head facing forward. This ensured that action sequences flowed naturally from clip to clip, as all clips started and ended with a neutral posture. Some of the triplets followed an *ABA* pattern (e.g., *turning head—raising leg—turning head*), and others followed an *ABB* pattern (e.g., *turning head—raising leg—raising leg*). Fig 1 contains frames excerpted from the human action clips used in both Experiment 1 and 2. They are the midpoint in time of each action clip, which depicts the maximum extent of movement.

**Fig 2:**
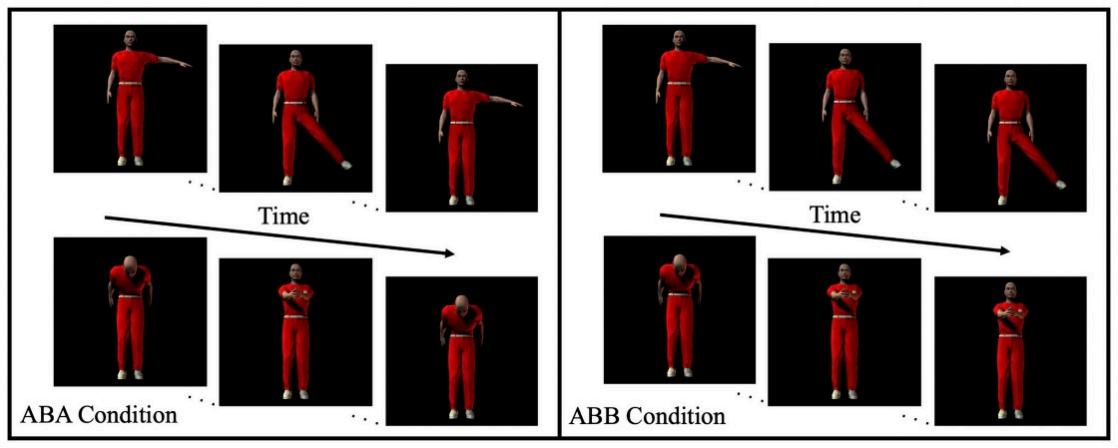
Examples of habituation materials from the ABA and ABB conditions in Experiment 1.

The habituation materials were created from eight unique human action clips, half assigned to class *A* and the other half assigned to class *B*. Then, the *A* and *B* action clips were combined exhaustively to create 16 unique *ABA* and 16 unique *ABB* human action triplets. Infants either saw *ABA* human action triplets (*ABA* habituation condition) or *ABB* human action triplets (*ABB* habituation condition) during habituation. Each habituation trial consisted of a different pseudo-random sequence of the 16 unique human action triplets, with each triplet lasting 1.8 seconds. Within a trial, triplets were separated by a 0.75-second blank screen to aid segmentation. The length of a given habituation trial was dependent on the infant’s looking behavior (see *Procedures*). Fig 2 shows the sample materials for each of the habituation conditions.

The test materials were comprised of four *completely novel* human action clips, two assigned to class *A* and two to class *B*. Four unique *ABA* action triplets and four unique *ABB* action triplets were generated from these four novel action clips. The test phase consisted of eight test trials, with each trial containing repetitions of a single action triplet, with a 0.75-second blank screen separating the repetitions. The number of repetitions for each infant varied, based on how long they looked at the stimuli. For each infant, half of the test trials followed the pattern seen during habituation (i.e., consistent), and half followed an inconsistent pattern (i.e., *ABB* pattern for infants habituated to the *ABA* pattern, and *ABA* pattern for infants habituated to the *ABB* pattern).

#### Procedures

We used a visual habituation procedure similar to those used in other experiments that tested for infants’ visual rule learning [15, 19]. The experiment consisted of a habituation phase and a test phase. The experiment started with an attention-getting video on the screen. As soon as the infant attended to the screen, the habituation phase started. A habituation trial consisted of a pseudo-random sequence of the 16 human action triplets. It began once the infant oriented to the screen and ended when the infant looked away from the screen for more than two consecutive seconds. The video looped if the trial was not terminated before the video reached the end. When a habituation trial ended, an attention-getting video appeared on the screen to recapture the infant’s attention before the next habituation trial started. An average looking time was calculated for every three non-overlapping habituation trials. (This departs from traditional habituation criteria that average over a moving window of habituation trials. This modification was accidental.) The habituation phase ended when the average of the infant’s looking times to the current three trials was less than 50% of the average looking time to the first three trials, or when the infant reached the maximum of 25 habituation trials (this never occurred).

The test phase started immediately after the habituation phase ended. During the test phase, all infants saw trials with consistent patterns and those with inconsistent patterns. The trial types alternated in the test phase, and the type of the first trial was counterbalanced across across infants. A test trial started once an infant attended to the screen and ended when the infant looked away from the screen for two consecutive seconds. If infants learned the non-adjacent rule (i.e., *ABA*) embedded in habituation, then we would expect them to look longer at the inconsistent test trials as compared to the consistent test trials.

### Results

**Fig 3:**
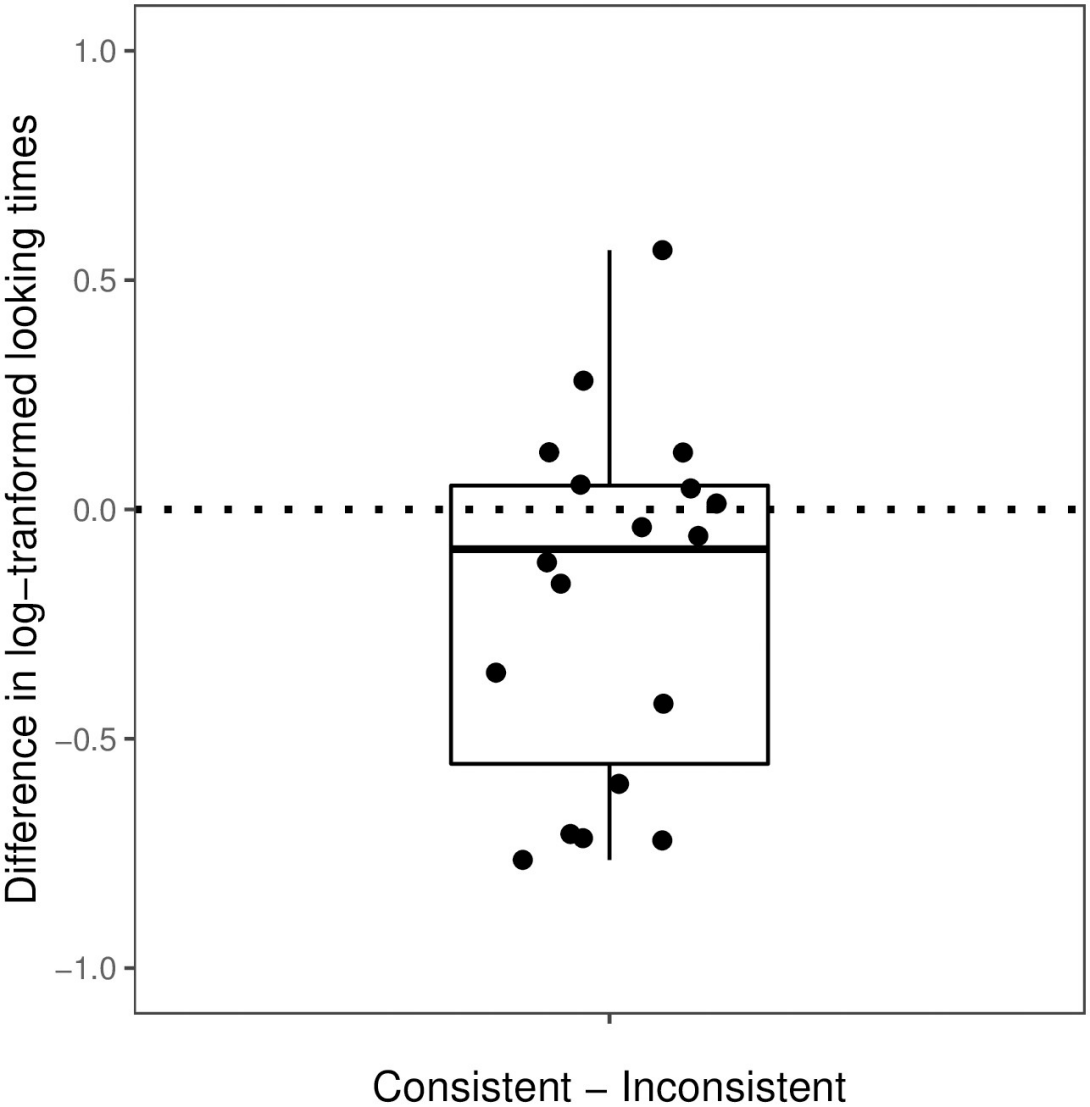
By-subject mean difference in log-transformed looking times between consistent and inconsistent test trials in Experiment 1.

We first excluded test trials with looking times less than 1.8 seconds (2 trials), because this was the time needed for seeing at least one iteration of the action triplet. We then log-transformed infants’ looking times, to account for the skew in looking-time data [19, 49]. For each infant, we then excluded individual trials that were outliers for that particular infant. A trial was identified as an outlier if log-transformed looking times were 1.5 times the interquartile range higher than the upper quartile or 1.5 times the interquartile range lower than the lower quartile for that infant (5 trials). Log looking times that were so deviant for a given subject were deemed to be unrepresentative of the underlying process of interest. (Analyses with outliers included were similar to the results we reported here, and are provided in the S1 File). After these steps, there were a total of 137 test trials from the 18 infants (144 subtracting 2 trials below 1.8 s threshold and 5 outliers). Each infant had at least six valid trials. For each infant, we then calculated their average log-transformed looking times to the consistent trials (*M_log_* = 9.06, *SD_log_* = 0.69) and their average log-transformed looking times to the inconsistent trials (*M_log_* = 9.27, *SD_log_* = 0.65). Fig 3 depicts the difference between the log-transformed looking times to the consistent and inconsistent trials for each infant.

To compare infants’ looking times, we ran a mixed effect linear regression, fitting main effects of *test consistency* (conforming to the habituation pattern or not), *test block* and *habituation condition*, and their interaction. The model also included by-subject random slopes for test consistency and test block. The main interest in the habituation condition and test block variables was to assess their interaction with test consistency. Habituation condition was included to see if there were differences due to the repetition pattern (*ABA* or *ABB*) seen in habituation, and test block was included to see if infants’ looking times change as a function of the number of test trials they had gone through. Each block contained one inconsistent trial and one consistent trial. All models reported in this paper were run in R Studio v1.3.959 [50] using the *lme4 v1.1.25* package [51] and the *lmerTest v3.1.3* package [52].

We found a significant main effect of test consistency such that infants looked longer to the inconsistent trials than to the consistent trials (*β* = 0.66, *SE* = 0.33, *p* = .046, see Table 1). The three-way interaction was not significant (*p* > 0.05). We did not find a significant main effect for habituation condition, and it did not interact with test consistency (all *p*’s > 0.05), showing that infants’ looking times to consistent and inconsistent trials were independent of which pattern they were habituated to. We also found two trending interactions: habituation condition x block (*p* = 0.098) and test consistency x block (*p* = 0.095). The interaction between habituation condition and block is hard to interpret and provides little information to our research question, as it does not involve test consistency, which is the variable related to learning. The trending interaction between test consistency and block suggests that as an infant went through more test trials, the difference between their looking times to the consistent and inconsistent trials was attenuated. This is consistent with findings in the infant literature that effects involving preference measures tend to diminish over time, as infants become familiar with both stimulus types (e.g., [19]).

**Table 1:** Summary of the fixed effects in the mixed effect linear regression model incorporating habituation condition, test consistency, block, and their interactions for Experiment 1.

Predictor	Coefficient	*SE*	*t*	*p*
Intercept	8.94	0.29	31.28	< 0.001***
Habituation	0.56	0.41	1.35	0.188
Consistency	0.66	0.33	2.02	0.046*
Block	0.06	0.09	0.61	0.547
Habituation x consistency	-0.61	0.46	-1.31	0.193
Habituation x block	-0.22	0.13	-1.69	0.098^†^
Consistency x block	-0.20	0.12	-1.68	0.095^†^
Habituation x consistency x block	0.26	0.17	1.59	0.115

*** *p* < 0.001;

* *p* < 0.05;

^†^ *p* < 0.10

Since the habituation condition did not interact with test consistency, we ran a new model that collapsed data from the two habituation conditions. We also removed the last block (i.e., the last consistent and the last inconsistent trial for each infant), given the weak evidence of an interaction in the context of a reasonable expectation of the attenuation of a test consistency effect. The new mixed effect linear model incorporates a main effect for test consistency with by-subject random intercepts and slopes for test consistency. We found a significant effect for test consistency: infants looked significantly longer to the inconsistent trials compared to the consistent trials (*β* = 0.28, *SE* = 0.11, *p* = .020, see Table 2).

**Table 2:** Summary of the fixed effects in the mixed effect linear regression model incorporating test consistency for Experiment 1, with test trials in the last block dropped.

Predictor	Coefficient	*SE*	*t*	*p*
Intercept	9.07	0.11	79.29	<0.001***
Consistency	0.28	0.11	2.56	0.020*

*** *p* < 0.001;

* *p* < 0.05

### Discussion

In this experiment, we tested 9-month-old infants’ ability to learn sequential repetition rules (i.e., *ABA* and *ABB*) from visual human actions. The results suggest that infants learned the visual sequential rule in the habituation phase and generalized it to the new test actions. This finding supports our prediction that dynamic human actions facilitate infants’ sequential rule learning in the visual domain. However, it contrasts with the findings of Johnson et al. [15], where 8- and 11-month-olds showed no evidence of learning when habituated to *ABA* sequences. It also contrasts with findings from rule learning studies involving human agents forming hand gestures [19], in conditions where the gestures were not demonstrated as being communicative.

What could account for the difference in learning outcomes between these various types of visual stimuli? We first consider differences between the stimuli we used here and static shapes [15]. One possibility is that the visual motion in the human action sequences were more engaging than the static shapes, and greater attention to the stimuli improved learning. It should be noted, however, that the shapes in Johnson et al.’s study [15] expanded in size as they were displayed, so there was a dynamic component in those stimuli as well. However, the dynamic component in the stimuli here involved transformations from one posture to another. Posture transformations involve more complex changes than size changes, as they involve changes in shape, not just metric properties, and this could make the dynamic component here more perceptually salient [53, 54]. In discussing a study with adults that used similar dynamic stimuli as those here, Lu & Mintz [42] propose that such transformations might highlight the temporal dimension for learners, and thereby focus learners’ attention on relationships across time, including non-adjacent relationships. (For a discussion of recent literature on the development of infants’ temporal orientation abilities and NAD learning, see [18].) Indeed, recent research investigating infants’ processing of human action sequences shows evidence that infants predict upcoming events in learned sequences [55].

It could also be that learning was facilitated because the human form in our experiment was a highly familiar object. That is, just as familiar stimuli might have facilitated rule learning in spatial arrays of dogs, cats, and faces [16, 17], the familiar human forms here could have resulted in better encoding of the stimuli. This, in turn, could have facilitated detecting the patterns over time, and maintaining them in memory.

Finally, it could be that the specific way human infants process visual information about human forms in action results in enhanced memory representations compared to other stimuli. As we mentioned earlier, recent studies show that processing human actions involve specialized brain regions, including motor cortex [44–46], compared to processing other visual information. This could result in stronger memory encoding and retrieval, which could in turn facilitate the detection of and memory for non-adjacent patterns. If so, then the advantage for human actions in visual rule learning would extend beyond the fact that they are familiar forms.

Turning to a comparison of our results to prior findings with stimuli of human agents forming ASL hand shapes from Rabagliati et al.’s study [19]. Recall that 7-month-olds failed to learn *ABA* rules, unless infants first saw the agent using the gestures in a communicative act with another agent and the infant. With pre-exposure that did not show a communicative act, or with no pre-exposure, infants failed to learn. Infants in our experiment presumably did not interpret the actions in Experiment 1 as communicative—at least, not any-more-so than infants in the unprimed conditions of Rabagliati et al.’s study—and so there is a contrast in results. One explanation is that infants in Rabagliati et al.’s study were younger, and rule learning is more challenging for younger infants. Infants in their communicative priming condition may have been more engaged with the stimuli—i.e., devoted greater attentional resources to it—which could have strengthened memory for the items and detection of the patterns. The distinguishing components of the gestures themselves were also more fine-grained than the movements in our stimuli. This could have made them perceptually less distinct. Representational differences between those stimuli and ours could also arise because their gestures generally involved just the arm, hand, and fingers, whereas our actions often involved larger movements of the torso, legs, arms, and head. If rule learning is indeed bolstered by human action stimuli in part because of involvement of the motor system in perception, and specialized areas for biological motion, then the grosser movements in our stimuli could resulted in greater activation of these systems and given an extra boost to learning in comparison to the gestures in Rabagliati et al.’s study. Under any of these possibilities, the communicative priming could have motivated infants to attend more to the stimuli, causing them to be more effectively encoded and facilitating the detection of non-adjacent patterns.

We further discuss the possible sources of improved visual-temporal *ABA* detection in the General Discussion. Regardless of the ultimate explanation, infants’ success at learning *ABA* repetition rules in Experiment 1 prompted us to further explore infants’ ability to generalize non-adjacent patterns from sequences of human actions.

## Experiment 2

Given infants’ success in learning *ABA* patterns in sequences of human actions, and given adults’ success in learning NADs from similar stimuli [40–42], we asked whether infants could learn NADs from sequences of human actions. We reasoned that, just as human actions supported *ABA* learning in the visual domain, compared to other stimuli [15], so might human actions support NAD learning where other stimuli do not, at least with 9-month-old infants [22, 23, 43]. To test this claim, in Experiment 2 we used visual human action sequences to examine 9-month-olds’ capacity to learn non-adjacent dependencies of the form *aXb*, where *a* and *b* each refer to a different specific item and *X* refers to one item from a class of items. Given the potential challenge of NAD learning in even younger infants, to provide the best chance of learning we included a pre-habituation phase that was intended to prime infants to the critical positions in an action triplet. The priming phase consisted of human action triplets that followed the *ABA* repetition pattern, similar to those seen in Experiment 1. By exposing infants to the *ABA* pattern first, we hoped that this would highlight relevance of the start and end positions of the triplets for infants, thus helping them better notice the non-adjacent dependencies embedded in the habituation sequences.

### Method

#### Participants

Infants were recruited from the same database used in Experiment 1. Parents were contacted by emails and phone calls. They gave informed consent before their child started the experiment. At the end of the experiment, we gave the child a t-shirt or a small toy as a token of appreciation. We tested 18 full-term infants between 8.5 and 9.9 months of age (*M* = 9.1 months) who did not participate in Experiment 1. Seven additional infants were tested but not included in the analysis due to fussiness (6) and exhibiting maximal looking times to more than half of the test trials (1). This was a predetermined exclusion criterion, with the rationale that with such long fixations across stimulus types, infants had not truly habituated in the habituation phase. In addition, when an infant ceilings out on multiple trials, any differences that would have emerged if the trials had been longer are suppressed. Nevertheless, we ran additional analyses that included this subject, and the overall outcomes were the same.

#### Apparatus

The experiment setup and the apparatus used were the same as in Experiment 1.

#### Stimuli

The experimental materials were human action video clips similar to those used in Experiment 1 (see Fig 1). Each video clip within a triplet lasted 0.6 second. Each clip started and ended with the animated human avatar in a neutral upright position with arms at the sides and head facing forward.

The priming phase consisted of *ABA* human action triplets similar to those used in Experiment 1. The priming materials were created from two class *A* human action clips and three class *B* human action clips. They were exhaustively combined to create six unique *ABA* human action triplets. Each priming trial consisted of a unique random order of the six triplets. Triplets within a priming trial were separated by a 0.75-second blank screen.

The habituation phase was comprised of ten novel human action clips that were not used in the priming videos. Four of the human action clips were used to create two NADs: *a_b* and *c_d* (here, each letter represents a unique human action clip). Of the remaining six clips, three (X_1–3_) occurred exhaustively in the middle position of *a_b*, and the other three (X_4–6_) occurred exhaustively in the middle position of *c_d* (creating a total of six unique triplets). Prior studies have shown that three intervening items provides sufficient variability for adults to learn the NADs in speech [30, 31] and in action sequences similar to these [42]. The assignment from action clips to the letters here were randomized for each infant, so that unintended similarities between the non-adjacent items—for example, actions both involve the hand—would not be confounded with the NADs. Each habituation trial had two blocks of the six randomly ordered triplets that were separated by 0.75-second blank screens.

The test phase contained eight different test triplets: four consistent triplets and four inconsistent triplets. Consistent triplets were of the form *aBb*, where *a_b* was attested in habituation (in comparison to *a_d*) and *B* represents one of the middle items (i.e., class B) that occurred in the priming phase. Inconsistent triplets were of the form *aBd*, combining the first item from one NAD triplet seen in habituation (e.g., *a* in *aX_1_b*), a middle (*B*) item from a priming triplet, and the last item from another habituation NAD triplet (e.g., *d* in *cX_5_d*). Thus, both consistent and inconsistent test triplets were novel and had zero adjacent transitional probabilities, but all the action clips within the triplets were familiar to infants, and in their familiar absolute positions. However, the consistent triplets followed the NAD patterns and the inconsistent triplets violated the patterns. As in Experiment 1, a given test trial consisted of repetitions of one action triplet.

**Fig 4:**
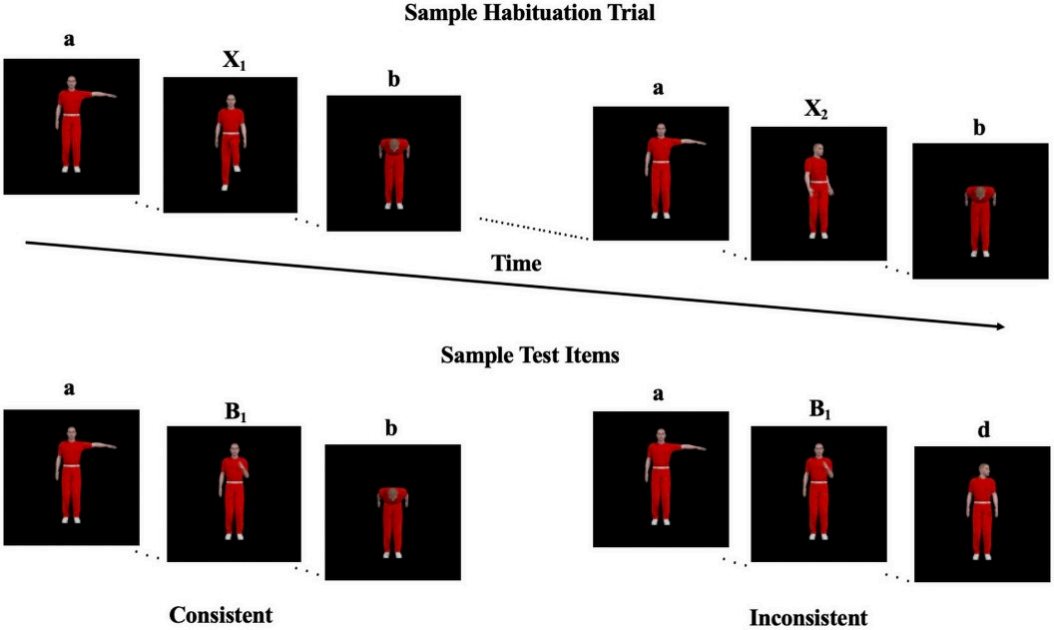
Examples of habituation materials and consistent vs. inconsistent test items in Experiment 2.

Fig 4 shows a sample habituation trial, and an example for each of the test item types.

#### Procedures

There were three phases in this experiment: a priming phase, a habituation phase, and a test phase.

The experiment started with an attention-getting video on the screen. As soon as the infant attended to the screen, the priming phase started. A priming trial started once an infant fixated on the screen, and ended when the infant looked away for two consecutive seconds or when the infant saw all 6 triplets in a trial (a total of 15.3 seconds). The priming phase ended when the infant reached a cumulative looking time of at least 30 seconds, or when the infant finished eight priming trials, whichever happened first. Since Experiment 1 showed that 9-month-old infants could learn the *ABA* pattern from human actions, exposure to these sequences could potentially highlight the non-adjacent relation and make it more salient for infants when they process the non-adjacent dependency triplets during the habituation phase.

The habituation phase followed the priming phase. A habituation trial started once the infant looked at the screen and ended when the infant looked away from the screen for more than two consecutive seconds, or when the infant saw all 12 triplets in that particular trial (a total of 30.6 seconds). When a habituation trial ended, an attention-getting video occurred to re-capture the infant’s attention. An average looking time was calculated for every three consecutive habituation trials (i.e., a moving window, rather than a blocked window as in Experiment 1). The habituation phase ended when the average of the infant’s looking times to the last three trials was less than 50% of the average looking time to the first three trials.

The test phase started once the infant met the habituation criterion. Infants saw both consistent and inconsistent test trials in the test phase, with trial types alternating within the test phase. The type of the first trial was counterbalanced across infants. A test trial started once the infant oriented to the screen, and ended when an infant looked away from the screen for two consecutive seconds, or when an infant saw six repetitions of the triplet. If infants are able to learn the NADs embedded in the habituation, then we expect them to look longer at the inconsistent triplets than the consistent triplets.

### Results

**Fig 5:**
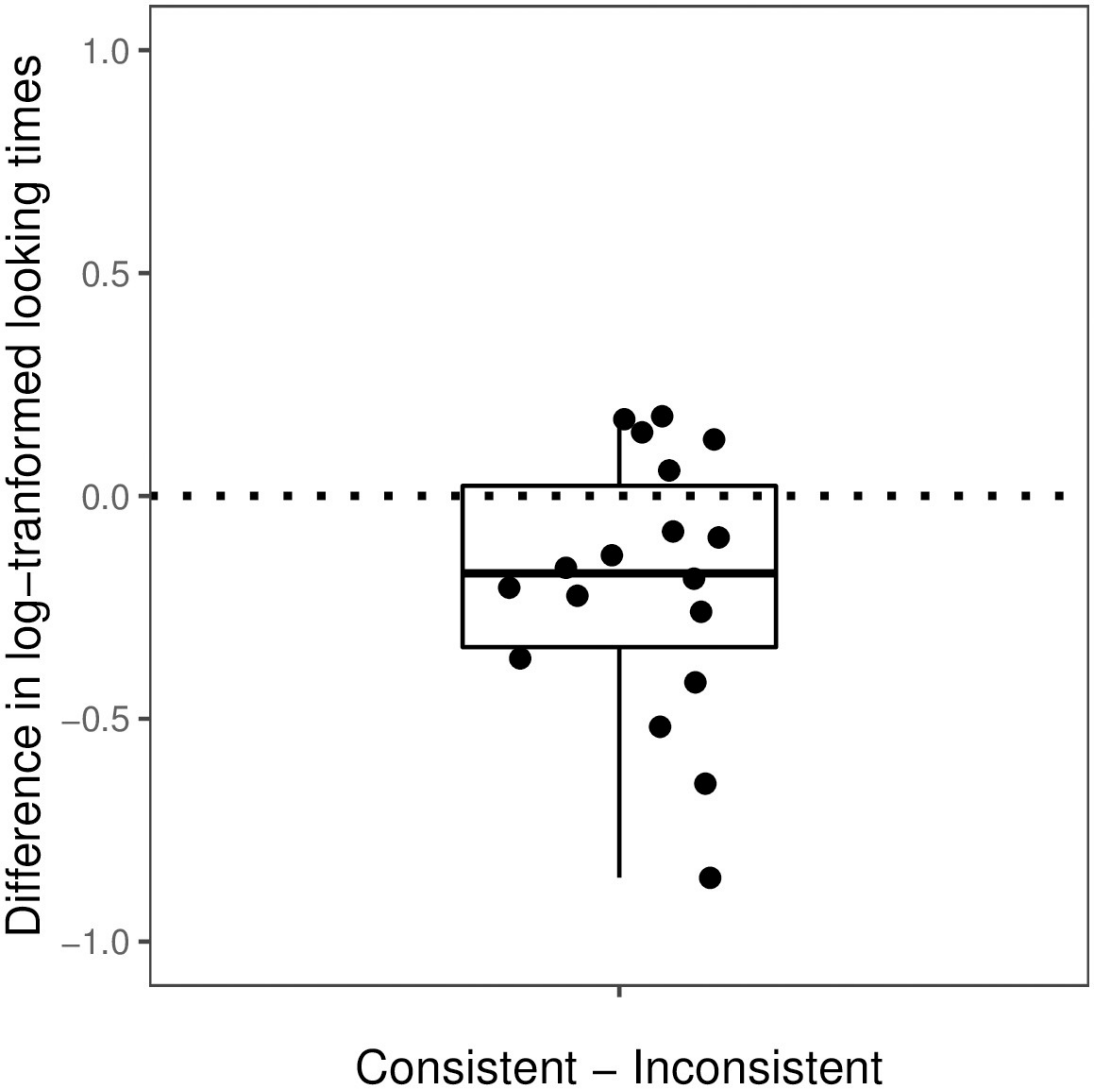
By-subject mean difference in log-transformed looking times between consistent and inconsistent test trials in Experiment 2.

As in Experiment 1, we first excluded trials with looking times that were shorter than 1.8 seconds (4 trials). We then log-transformed the data and removed outlier trials for each infant, as described in the Results section of Experiment 1 (8 trials). After these steps, we had a total of 132 test trials from the 18 infants, and each infant had at least six valid trials. For each infant, we calculated their average log-transformed looking times to the consistent trials (*M_log_* = 8.70, *SD_log_* = 0.46) and their average log-transformed looking times to the inconsistent trials (*M_log_* = 8.90, *SD_log_* = 0.49, see Fig 5 for the differences between the log-transformed looking times to the consistent and inconsistent trials).

To compare infants’ looking times, we ran a mixed effect linear regression, fitting main effects of *test consistency* (conforming to the habituation NADs or not), *test block*, and their interaction, with by-subject random intercepts—this was the maximal model that converged. As shown in Table 3, we did not find a significant main effect for block (*p* = 0.569) nor an interaction between test consistency and block (*p* = 0.325). Because no significant effect was found for block or its interaction with test consistency, we ran another model with a fixed effect of test consistency only and by-subject random intercepts for test consistency—including random slopes elicited a singularity warning. There was a significant effect of consistency, indicating that infants looked significantly longer to the inconsistent trials (*β* = 0.20, *SE* = 0.07, *p* = 0.006, see Table 4), as in Experiment 1.

**Table 3:** Summary of the fixed effects in the mixed effect linear regression model incorporating test consistency, block, and their interaction for Experiment 2.

Predictor	Coefficient	*SE*	*t*	*p*
Intercept	8.76	0.13	65.13	<0.001***
Consistency	0.36	0.17	2.07	0.041*
Block	-0.03	0.05	-0.57	0.569
Consistency x block	-0.06	0.06	-0.99	0.325

*** *p* < 0.001;

* *p* < 0.05

**Table 4:** Summary of the fixed effects in the mixed effect linear regression model incorporating only test consistency for Experiment 2.

Predictor	Coefficient	*SE*	*t*	*p*
Intercept	8.70	0.07	116.20	<0.001***
Consistency	0.20	0.07	2.79	0.006**

*** *p* < 0.001;

** *p* < 0.01

### Discussion

In this experiment, we found that 9-month-old infants could learn NADs from visual human actions, if they had a brief exposure to a non-adjacent *ABA* repetition rule first. This is the first report that we are aware of to show item-specific NAD learning for visual stimuli in 9-month-old infants. Moreover, infants learned the NAD patterns when the intervening position varied between only three items. This contrasts with some findings in the speech domain that suggest that learners require greater variability in the medial items in order to detect the NADs [21, 22]; though it is consistent with more recent studies that have shown that at least adults can learn NADs with only three different intervening elements [30, 31]. The results also contrast with the results for 9- to 12-month-old infants in Bettoni et al.’s study [43], where subjects did not show evidence of discriminating consistent versus inconsistent test trials. As we mentioned earlier, the dependent items in that study were geometric shapes, and the intervening items were arrays of dots. Hence, there was a strong perceptual similarity between the dependent items in that study, and they differed perceptually from the intervening ones, as in the studies with non-linguistic auditory materials [33, 34]. In contrast, the stimuli here were all of the same type, affording no surface level cues to link the non-adjacent items. In addition, a feature of Bettoni et al.’s study, as well as of many studies of NAD learning in speech [21, 22, 27, 31], is that the consistent test items were actually identical to the habituation items. In contrast, the consistent test items in Experiment 2 were novel triplets, since the medial position was always filled with an action from the priming phase, so infants had to generalize the NAD pattern to sequences with novel medial items. All of these stimulus differences arguably made the stimuli here more challenging for detecting NADs, yet the 9-month-olds successfully learned and generalized the patterns.

There are two properties of our stimuli that could have facilitated learning. First, the priming phase may have had its intended effect of guiding learners’ attention to the relationship between the dependent items at the edges of the action triplets. If so, we cannot say whether or not this was critical to infants’ success in learning the NADs. We speculate that even if it was necessary, it may not have been sufficient to support learning; after all, in Bettoni et al.’s study [43] where the NADs themselves were perceptually similar, and differed from the intervening items, 9-month-olds nevertheless did not learn the NADs. That was arguably a much more direct kind of cue than priming in Experiment 2. Perhaps a larger influence was the second property: that the stimuli were human actions. As outlined in the discussion to Experiment 1, the dynamic transformations could have heightened the salience of the temporal dimension thereby facilitating the detection of the dependencies over time. The familiar human forms could have resulted in stronger memory representations, also facilitating the processing necessary to detect the dependencies. Finally, memory could be facilitated by the recruitment of motor cortical areas [44] and areas specific to processing biological motion [45, 46] as a part of the process of perceiving human forms in action.

It is interesting to note that there is less variability in the estimate of the effect of test item consistency in Experiment 2 compared to Experiment 1 (see Tables 2 & 4), indicating a more reliable effect. It seems unlikely that NADs would be easier to learn than repetition rules; this effect could be the result of the priming phase. A replication of these experiments could determine whether this difference is reliable.

The data from this experiment provide no evidence about the specific mechanisms that account for the apparent advantage of human actions over other visual stimuli in supporting NAD learning. The possibilities just outlined are currently only speculations. But the evidence does show that, when primed with a non-adjacent repetition rule, 9-month-old infants can learn NADs in visual sequences of human actions.

## General discussion

The findings from Experiment 1 demonstrate that 9-month-old infants can learn repetition rules, including non-adjacent, *ABA* rules, in non-communicative, temporally sequential visual stimuli. In Experiment 2, 9-month-olds were primed with *ABA* stimuli, then exposed to visual human actions sequences containing NADs, and infants learned the NADs. These two experiments show that 9-month-old infants have the capacity to learn two different types of non-adjacent dependencies in temporally sequenced stimuli. The findings contrast with those from prior studies that used different types of visual stimuli [15, 19, 43], indicating that stimulus differences are likely to be substantially responsible for the differences in results.

### Stimulus advantages for human actions

In the discussion sections of the experiments, we outlined several mutually tenable hypothesis about the mechanisms by which human action stimuli could provide greater support for learning non-adjacent patterns compared to other stimuli. These were, a) focusing attention to the temporal dimension of the stimuli, over which the non-adjacent patterns hold [42], b) enhancing memory representations due to the familiarity of the human form [16], and c) the recruitment of motor cortex [44] and areas specialized for processing biological motion [45, 46] when perceiving human action. The candidate mechanisms each facilitate the detection of and memory for non-adjacent patterns, either by enhancing the memory representations, or focusing attention to the temporal dimension of the stimuli.

**Fig 6:**
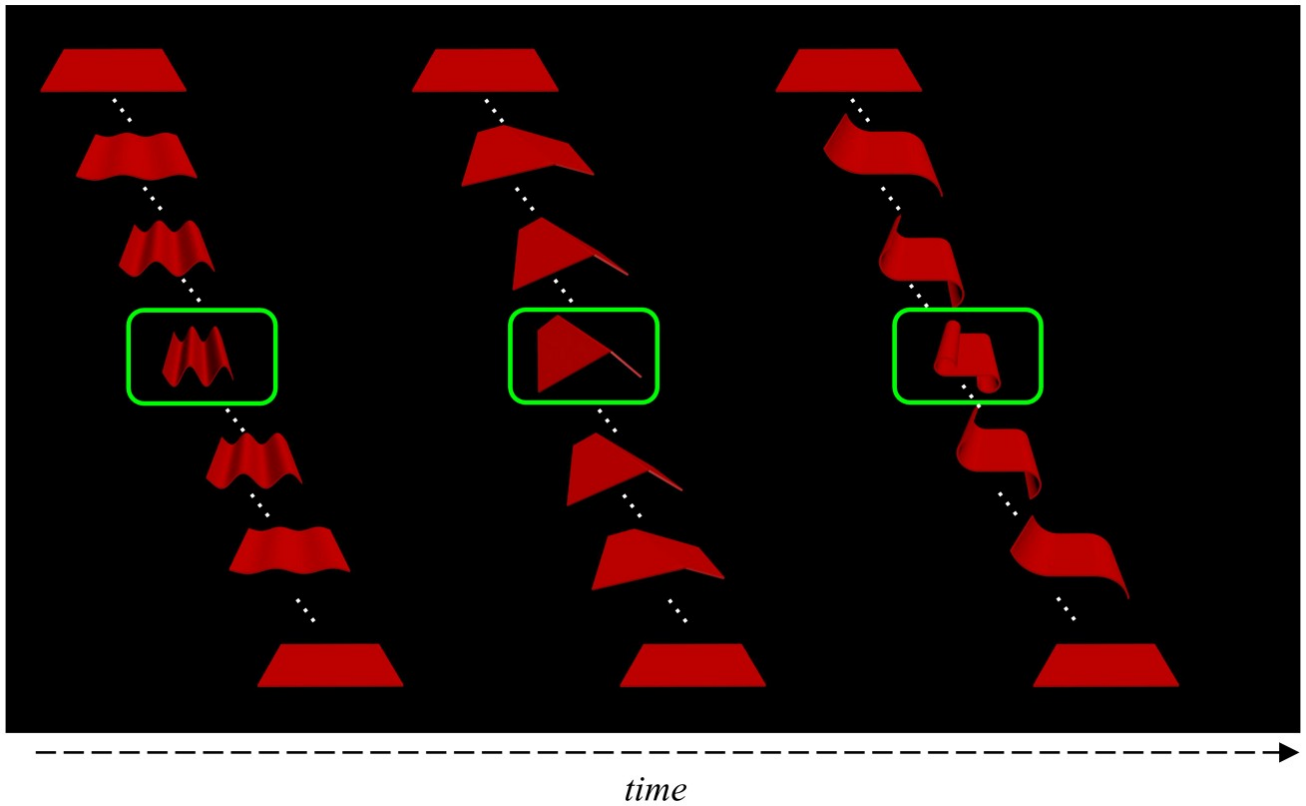
Example of an object transformation triplet from [41]. The images depict a sample of frames in a continuous video of an object—the red plane—transforming into each of three different target shapes, marked here with a green boundary, and back to its original form. In the video, the dynamic object was shown in the center of the display, whereas here the spatial arrangement from left to right depicts time. The duration of each transformation was the same; the figure does not depict the durations to scale. In one version of the experiment, the static images depicted in the boxes were displayed instead of its associated video clip.

There is some evidence that the property of dynamic transformations is sufficient for supporting visual NAD learning, at least in adults. When Li and Mintz [41] replaced each human action video with a different video of an object (a red 3-dimensional plane) transforming into a different shape (e.g., by twisting, folding, etc.), adults learned NADs of object transformation sequences as well, but not of static object images (Fig 6). Similarly, Lu and Mintz [42] found that adults successfully learned NADs from visually presented dynamic human actions, but not with static human postures, with the same amount of training. So, at least with adults and NAD learning, dynamic stimuli appear to engage learning mechanisms more than static stimuli do. Testing infants on similar materials will shed light on the contribution of the dynamic aspect of the stimuli used here to support non-adjacent pattern learning.

Another consideration in understanding infants’ success in learning NADs in Experiment 2 is that they were exposed to *ABA* sequences in the priming phase. This design feature was intended to focus their attention to the relevant position for the the NADs in the subsequent action triplets. It may be that this priming was critical to 9-month-olds’ ability to learn the NADs. If so, it would be further evidence of the challenges of NAD learning for infants, but it would also indicate where the challenges lie. If priming infants to attend to the dependent positions is necessary, it suggests that infants’ difficulty may be in detecting the NADs, not not necessarily in remembering the dependent items. We are planning to address this question in future studies. It also remains to be seen whether priming in the auditory domain could facilitate NAD learning. Preliminary experiments from our lab have not found evidence of NAD learning in an artificial language in 9-month-olds, even with *ABA* priming, but this is still an open question.

### Implications for language acquisition

Aside from being a positive demonstration of visual NAD learning in infants, the results from Experiment 2 also have implications for language acquisition. As noted earlier, infants younger than 12 to 15 months appear to have difficulty learning NADs in spoken artificial languages, at least when measured behaviorally. Yet, the neurophysiological data suggest that, at some level, infants process NADs as young as three months [27]. Those data, combined with the present findings, indicate that pre-lingual infants have the computational capacity to learn NADs across multiple domains. The prior difficulty in eliciting behavioral evidence of NAD learning in younger infants in the speech domain might, then, be a result of the capacity of the stimuli to generate richly encoded memory representations. This potential deficit might arise more in NAD learning than *ABA* learning, for the following reasons. In *ABA* learning, infants must maintain item specific memory for the first *A* long enough to detect a repetition when encountering the second one. However, when a repetition rule is extracted the specific items no longer need to be remembered for the rule to be ‘applied’ when processing subsequent sequences. In contrast, infants must remember the specific items (i.e., the specific relationship between *a* and *b* in an *a_b* frame) across the habituation period and into the test phase. It is possible that the dynamic visual actions used in Experiment 2 result in more lasting, and perhaps more easily related representations than speech. If true, this would be surprising. After all, speech is an important ecological signal, and there is strong evidence that infants’ attention is drawn to speech from very early in development [56, 57]. Yet, our particular visual stimuli depicting conspecifics are also ecologically important [58], and humans in motion may be especially good at capturing attention [59, 60]. For behavioral experiments of relatively short duration and with somewhat unnatural exposure conditions—disembodied voices in speech studies, entities on a 2D display in visual studies—there could plausibly be an advantage for dynamic human action over speech. We are currently planning studies that combine speech and dynamic human action to test whether multimodal stimuli facilitate the acquisition of NADs in speech.

Behavioral differences in learning clearly do arise from differences in stimuli in NAD experiments with infants, whatever the reason(s) may be. The apparent advantage for visual human actions is notable, given what we know about infants’ attraction to speech. Nevertheless, Experiment 2 does provide us with clear evidence from a behavioral study that pre-lingual infants can learn NADs—consistent with findings using neurophysiological measures [27]—and we have hypothesized possible reasons for the differences in different stimuli’s ability to elicit learning. With respect to understanding when infants can incorporate processing NADs into processes of spoken language acquisition, it will be important to investigate whether even slightly more naturalistic learning scenarios—for example with mulitmodal visual input of the talker, as alluded to earlier—support NAD learning. This will help determine when theories can assume learners have access to information about non-adjacent relationships to support language acquisition.

The previous discussion raises the question of whether the mechanisms of NAD learning across domains are the same. Attempts to explain behavioral differences in terms of different stimulus properties, as opposed to intrinsic differences in the stimuli, makes the implicit assumption that they are. However, recent research with 2- and 3-year-olds in the auditory domain suggest that processing NADs in speech and non-speech sounds follows different developmental trajectories, and is supported by different brain regions [61]. If speech is indeed special with respect to NAD learning, then insights gained from studying NAD learning in the visual domain may have limited application to understanding NAD learning in speech. Nevertheless, the perception of speech and of visual human action do have commonalities, in that they both involve perceiving the consequence of human action. It could turn out that, in the visual domain, human action is processed differently than other visual sequences. Our results are consistent with that possibility, since we found evidence of learning two types of non-adjacent patterns in human actions that have not been observed in sequences of shapes, at least in 9-month-olds. An intriguing, but entirely speculative possibility is that learning patterns in speech and visual sequences of human actions may have more in common than learning in other types of stimuli within the auditory and visual domains.

## Conclusion

In the two experiments in this study, we used sequences of dynamic human action to investigate 9-month-old infants’ ability to learn two different types of patterns involving non-adjacent items. The results from Experiment 1 showed that infants can learn non-adjacent repetition rules (*ABA* patterns) and apply them to instances of new actions. Results from Experiment 2 demonstrated that infants can learn item-specific non-adjacent dependencies and generalize to the new sequences that maintain the NAD, but with novel intervening items, at least when primed with *ABA* patterns. This is the youngest age at which a behavioral experiment has found evidence of NAD learning. In both experiments, we proposed that the stimuli, dynamic human actions, provided a more robust signal than those used in experiments that failed to show evidence of learning. At the moment, we can only speculate as to the mechanisms that make human action sequences such a supportive signal. Whatever the ultimate explanation, this study shows that 9-month-olds can learn non-adjacent repetition rules and item-dependent non-adjacent dependencies in sequences of visual human actions.

## Supporting information

S1 File Model outcomes with data including outlier trials. This supporting information includes tables presenting results from the models reported in Experiment 1 and 2, with the outlier trials included.
(DOCX)
