# Supplementary material for: Learning non-adjacent rules and non-adjacent dependencies from human actions in 9-month-old infants
Source: PLoS One. 2021 Jun 9;16(6):e0252959. doi: 10.1371/journal.pone.0252959 (PMC8189460; doi:10.1371/journal.pone.0252959)
Supplement: S1 File — This supporting information includes tables presenting results from the models reported in Experiment 1 and 2, with the outlier trials included. (DOCX) [file pone.0252959.s001.docx]

S1 File. Model outcomes with data including outlier trials.

**Experiment 1**

S1 Table 1

*Summary of the fixed effects in the mixed effect linear regression model incorporating habituation condition, test consistency, block, and their interactions for Experiment 1, including outlier trials.*

| Predictor | Coefficient | *SE* | *t* | *p* |
| --- | --- | --- | --- | --- |
| Intercept | 8.94 | 0.27 | 32.52 | <0.001 |
| Habituation | 0.15 | 0.39 | 0.38 | 0.703 |
| Consistency | 0.71 | 0.37 | 1.95 | 0.054 |
| Block | 0.06 | 0.09 | 0.62 | 0.537 |
| Habituation x consistency | -0.15 | 0.51 | -0.29 | 0.772 |
| Habituation x block | -0.10 | 0.13 | -0.76 | 0.452 |
| Consistency x block | -0.23 | 0.13 | -1.71 | 0.089 |
| Habituation x consistency x block | 0.15 | 0.19 | 0.81 | 0.420 |

S1 Table 2

*Summary of the fixed effects in the mixed effect linear regression model incorporating test consistency for Experiment 1, including outlier trials and excluding test trials in the last block.*

| Predictor | Coefficient | *SE* | *t* | *p* |
| --- | --- | --- | --- | --- |
| Intercept | 9.02 | 0.12 | 76.51 | <0.001 |
| Consistency | 0.35 | 0.12 | 2.87 | 0.005 |

**Experiment 2**

S1 Table 3

*Summary of the fixed effects in the mixed effect linear regression model incorporating test consistency, block, and their interaction for Experiment 2, including outlier trials.*

| Predictor | Coefficient | *SE* | *t* | *p* |
| --- | --- | --- | --- | --- |
| Intercept | 8.91 | 0.14 | 62.21 | <0.001 |
| Consistency | 0.29 | 0.20 | 1.50 | 0.136 |
| Block | -0.04 | 0.05 | -0.87 | 0.385 |
| Consistency x block | -0.05 | 0.07 | -0.75 | 0.455 |

S1 Table 4

*Summary of the fixed effects in the mixed effect linear regression model incorporating test consistency for Experiment 2, including outlier trials.*

| Predictor | Coefficient | *SE* | *t* | *p* |
| --- | --- | --- | --- | --- |
| Intercept | 8.70 | 0.07 | 131.56 | <0.001 |
| Consistency | 0.16 | 0.08 | 1.97 | 0.051 |
